# Efficient lysis of B-chronic lymphocytic leukemia cells by the plant-derived sesquiterpene alcohol α-bisabolol, a dual proapoptotic and antiautophagic agent

**DOI:** 10.18632/oncotarget.25398

**Published:** 2018-05-25

**Authors:** Antonella Rigo, Isacco Ferrarini, Angela Bonalumi, Cristina Tecchio, Alessio Montresor, Carlo Laudanna, Fabrizio Vinante

**Affiliations:** ^1^ Section of Hematology, Department of Medicine, University of Verona, Verona, Italy; ^2^ Cancer Research and Cell Biology Laboratory, Department of Medicine, University of Verona, Verona, Italy; ^3^ Section of General Pathology, Department of Medicine, University of Verona, Verona, Italy

**Keywords:** apoptosis, autophagy, chronic lymphocytic leukemia, treatment, α-bisabolol

## Abstract

The sesquiterpene α-bisabolol (α-BSB) is a cytotoxic agent against acute leukemia and chronic myeloid leukemia cells. Here the profile of α-BSB citotoxicity was evaluated *ex vivo* in primary mononuclear blood cells isolated from 45 untreated B-chronic lymphocytic leukemia (B-CLL) patients. We studied the effects of α-BSB by flow cytometric and western blotting techniques with the following findings: (1) α-BSB was an effective proapoptotic agent against B-CLL cells (IC_50_ 42 ± 15 μM). It was also active, but to a lesser extent, on normal residual B cells and monocytes (IC_50_ 68 ± 34 and 74 ± 28 μM, respectively; *p* < 0.01), while T-cells, though not achieving IC_50_, were nevertheless decreased. (2) Lipid raft content positively correlated with α-BSB cell sensitivity, while neither the phenotype of B-CLL cells nor the disease clinical stage did affect the sensitivity to α-BSB. (3) Flow cytometry analysis evidenced the induction of pores in mitochondrial and lysosomal membrane after 3- to 5-hour exposure of B-CLL cells to α-BSB, leading to apoptosis; in contrast, western blotting analysis showed inhibition of the autophagic flux. Therefore, according to cellular selectivity, α-BSB is a cytotoxic agent preferentially active against leukemic cells, while its lower activity on normal B cells, monocytes and T cells may account for an additive anti-inflammatory effect targeting the leukemia-associated pro-inflammatory microenvironment. Consistent with the observed effects on intracellular processes, α-BSB should be regarded as a dual agent, both activating mitochondrial-based apoptosis and inhibiting autophagy by disrupting lysosomes.

## INTRODUCTION

B chronic lymphocytic leukemia (B-CLL) is the commonest malignancy in the western world. Usually an indolent disease that primarily affects elderly adults, it is characterized by monoclonal CD5^+^ mature B cells, which accumulate in bone marrow, lymphoid tissues and blood and are mostly blocked in G_0_/G_1_ phase of the cell cycle [1, 2]. Some patients may experience a more aggressive course: secondary hemolytic anemia and/or autoimmune thrombocytopenia may develop, and malignant progression may be seen. Eventually even the most indolent B-CLL leads to bone marrow insufficiency and symptomatic splenomegaly due to neoplastic substitution.

Over the last twenty years, a deep revision of its pathogenesis has thoroughly changed the understanding of B-CLL as well as its clinical and therapeutic management [3]. The turning-points in this progress can be summarized as follows. *(a)* Recognition of molecular rearrangements defining more aggressive courses, namely the somatic mutational status [2, 4–7] and chromosomal alterations such as del11 and 17 [3, 8]. *(b)* Understanding of immunoglobulin B-cell receptor [6, 9] and microenvironment-driven signals [10–20] pathogenetic potential. *(c)* Better understanding of kinase-based transduction [10, 11, 21], programmed cell death and autophagy pathways as well as their gene regulation [14, 19, 22, 23]. *(d)* Availability of new agents (including anti-CD20 and other monoclonal antibodies as well as several kinase inhibitors) that turned out to be more effective than ever before, though perhaps not yet sufficient to cure the disease [24–27]. A better knowledge of the enzymatic pathways regulating adhesion signaling, apoptosis and autophagy as well as related oncogenes has pioneered and fostered the design of and the quest for therapies targeted at components of those pathways. The other way round, the differential efficacy of several of these targeted therapies has shed light on some more relevant mechanisms and transduction pathways in B-CLL [11, 25, 27]. New agents for targeted therapy include specific protein kinase inhibitors, apoptosis activators and autophagy inhibitors, some of them man-projected, some others sorted through existing cellular organic molecules. [3, 4, 9, 19, 24]. Among these latter, sesquiterpenes [28] like artemisinin [29] gossypol [30] or α-bisabolol (α-BSB) [31] may have practical and theoretical relevance to B-CLL therapy. These multifaceted, virtually limitless, target-restricted therapies should be in compliance with some traditional cornerstones for B-CLL treatment: *(a)* low toxicity; *(b)* easily administration preferably orally or subcutaneously; *(c)* good specificity for the therapeutic targets; *(d)* opportunity for synergism. In this context, the sesquiterpene alcohol α-BSB is safe at the therapeutic dosages in animal models, can be delivered orally [31], targets specific basic cellular functions like apoptosis and autophagy [22] and is synergistic with some tyrosin kinase inhibitors [32]. Here, we investigated the antineoplastic potential of α-BSB in a preclinical model of primary normal and neoplastic cells from untreated B-CLL patients.

## RESULTS

### Patients

Table 1 summarizes the main characteristics of the 45 untreated patients who underwent evaluation. They were diagnosed with B-CLL starting from 2002. The great majority were Ig-mutated, normal-caryotype, Binet A-stage males with more than 47 years, more than 12 × 10^−9^/L white blood cells (WBC) and 100 × 10^−9^/L platelets (PLTs), without anemia. We could not establish in this study any different sensitivity to α-BSB related to clinical stage according to Binet or to biological characteristics of B-CLL cells (i.e. CD38 positivity, IgVH mutational state or chromosomal abnormalities).

**Table 1:** Patients' main clinical characteristics

Sex M/F	Age^a^	Hb^a^ g/L	WBC^a^ 10^−9^ /L N%-L%	PLTs^a^ 10^−9^/L	IgVH UnM/M/ne/nd^b^	FISH Normal/+12/del(13)/ ne^b^	CD38Neg/Pos	Binet A/B-C
33/12	69.5 (47–83)	145 (111–174)	24 (12.1–90) 5 (3–12.7)–75 (50–90)	215(101–400)	14/23/4/4	26/6/10/3	36/9	32/13

^a^median & range.

^b^UnM = unmutated; M = mutated; ne = not evaluable; nv = not done.

### α-BSB inhibits B-CLL cell viability

**Figure 1:**
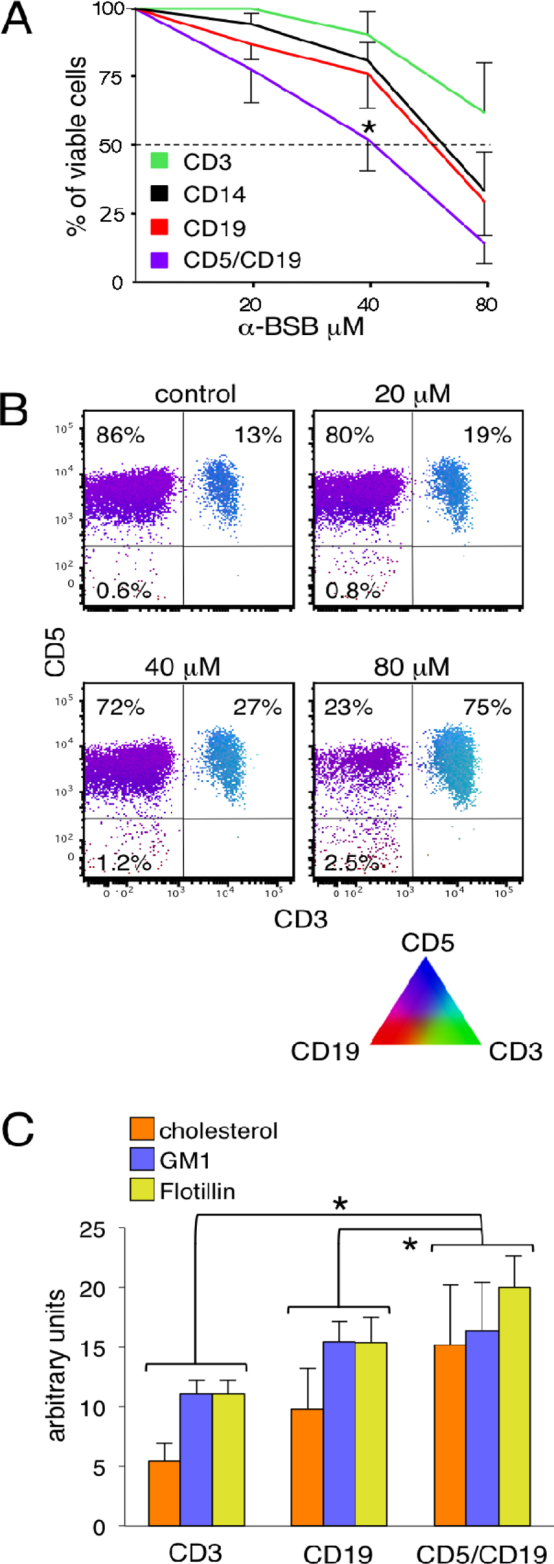
α-BSB reduced B-CLL cell viability (**A**) Cytotoxicity assay. PBMC of 45 patients were treated with α-BSB at the indicated concentrations for 24 hours. Sensitivity to α-BSB was significantly different between B-CLL lymphocytes and normal T and B cells and monocytes (^*^p = 0.005). Results are expressed as mean ± SD. (**B**) PolyChromatic plot analysis on treated CD5/CD19 B-CLL lymphocytes as compared to normal T and CD19 B cells. PBMC from a B-CLL representative case with 13% of normal T lymphocytes were treated with 20, 40, 80 μM α-BSB for 24 hours, then immunostained with anti-CD3-FITC, anti-CD5-APC and anti-CD19-PE moAbs and analyzed by flow cytometry. The triangle display illustrates the different dummy colors assigned to each marker and the mixtures that result by their different amounts. The bivariate PolyChromatic plots shows the color mapping results: the subsets of cells can be identified by the color scheme. CD5/CD19 B-CLL cells are magenta, CD19 normal B cells are red, CD5/CD3 T cells are turquoise. The comparison of the plots gives evidence that α-BSB induced a dose-dependent preferential depletion of B-CLL lymphocytes. (**C**) Lipid rafts in normal T, CD19 B cells and in CD5/CD19 leukemic cells. PBMC of 45 patients were prestained with anti-CD3-PerCP, CD5-APC and anti-CD19-APC-H7 moAbs. Cholesterol, GM1 and Flotillin-1 contents were determined by flow cytometry and expressed as arbitrary units. Overall, they were less abundant in T cells than in residual normal CD19 B cells than in CD5/CD19 B-CLL cells (^*^p < 0.05 for each comparison), though the single GM1 component was only slightly increased in leukemic B-CLL cells. Means ± SD of 7 experiments are shown.

For each patient, we comparatively evaluated the *ex vivo* sensitivity to α-BSB of B-CLL cells, normal residual B, T lymphocytes and monocytes. 2 to 80 μM α-BSB for 24 hours resulted in a dose-dependent reduction of B-CLL cell viability. Figure 1A shows that leukemic lymphocytes IC_50_ was 42 ± 15 μM α-BSB, significantly lower than 68 ± 14 and 72 ± 12 μM α-BSB of normal B cells and monocytes, respectively (*p* = 0.005). Instead, T lymphocytes did not reach the IC_50_ in the range of concentrations tested (up to 80 μM α-BSB), and were considered minimally sensitive to α-BSB. Accordingly, Figure 1B shows the preferential depletion of CD5/CD19 B-CLL cells as opposed to the normal mononuclear ones. Thus, α-BSB inhibited leukemic cell viability in a dose-dependent manner at concentrations also affecting residual normal B lymphocytes, monocytes and at a lesser extent normal T cells.

### Lipid raft amounts

At the cell membrane level, α-BSB accumulates in the lipid rafts (LRs) [33]. Therefore, we sought if the different sensitivity to α-BSB observed in leukemic as compared to normal B lymphocytes could be related to a far more represented LRs in neoplastic cells than in their normal counterparts [34]. The largest components of LRs are glycosphingolipids such as ganglioside monosialic 1 (GM1), cholesterol, sphingomielin and phosphatidyl serine. GM1 is a receptor for toxins and viral particles [35, 36] whose amounts have been demonstrated to correlate with the response to anti-CD20 therapy in B non-Hodgkin lymphomas and B-CLL [37]. Flotillins are another LR component belonging to a family of integral membrane proteins that are actively involved in signal transduction as well as in regulating the motility and localization of LRs [38]. Both Flotillin-1 and Flotillin-2 are commonly used as LRs associated markers [39–41]. In B-CLL cells and in normal B and T lymphocytes of the same sample we quantified cholesterol, GM1 and Flotillin-1 by flow cytometry. Cholesterol was quantified by its affinity to the polyene antibiotic filipin which has fluorescence properties, GM1 by means of its affinity to cholera toxin B (CT-B), and Flotillin-1 by a specific monoclonal antibody [42]. As shown in Figure 1C, lipid raft amounts resulted significantly more abundant in CD5/CD19 B-CLL cells than in normal T and CD19 B cells (*p* < 0.05 for each comparison). Altogether, these findings correlated with the different sensitivity to α-BSB seen in the different cell subpopulations, accordingly to previous suggestions [33].

### α-BSB causes damage to plasma membrane

**Figure 2:**
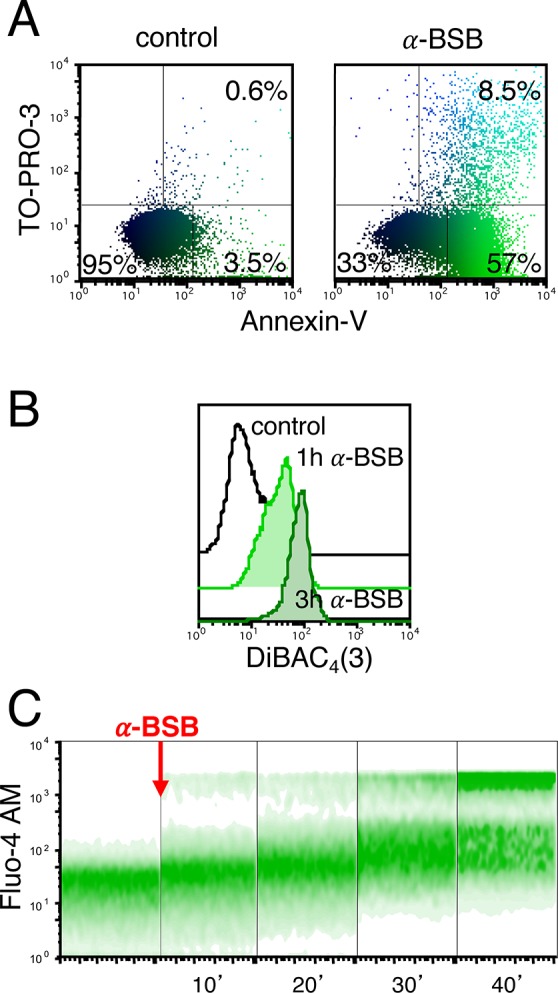
α-BSB caused rapid loss of plasma membrane integrity in B-CLL cells Treatment with 80 μM α-BSB for 1 and 3 hours. (**A**) PBMC stained with Annexin-V and TO-PRO-3 after treatment. Distinction between unaffected cells (AnxV-neg/TO-PRO-3-neg, mean percentages 95.2 ± 1.9_control_ versus 31.6 ± 4.2_treated_), early apoptosis (AnxV-pos/TO-PRO-3-neg, mean percentages 3.8 ± 1.1_control_ versus 57.4 ± 5.2_treated_) and late apoptosis (AnxV-pos/TO-PRO-pos, mean percentages 0.5 ± 0.3_control_ versus 9.7 ± 1.7_treated_). (**B**) Time-dependent increase of cell fluorescence after cell incubation with α-BSB and DiBAC_4_(3) anionic dye, indicating the depolarization of the plasma membrane. (**C**) Density plot data showed the increase of intracellular Ca^2+^ concentration revealed by the Ca^2+^ indicator Fluo-4 AM starting from 10 to 40 minutes after treatment, thus confirming the rapid loss of homeostatic plasma membrane impermeability. Each shown experiment is representative of 5.

To investigate the chain of events leading to the loss of cell viability, we initially stained the B-CLL cells with TO-PRO-3 iodide, which binds with high affinity to double-strand nucleic acids but does not enter intact plasma membrane, and Annexin-V to determine the lipid phosphatidylserine flip from the inner to the outer leaflet of the plasma membrane. We observed a time-dependent increase (data not shown) of TO-PRO-3 and Annexin-V fluorescence when cells were treated with 80 μM α-BSB (Figure 2A). After 3 hours of incubation with α-bisabolol, a substantial proportion of B-CLL cells were Annexin-V-positive, thus signaling the irreversible onset of the apoptotic cascade. Then, to assess the depolarization of plasma membrane upon α-BSB, we used the sensitive anionic dye [Bis-(1,3-Dibutylbarbituric Acid)Trimethin Oxonol] [DiBAC_4_(3)], a slow-response probe that enters the depolarized membrane, with an increase in cell fluorescence. The mean fluorescence intensity (MFI) of DiBAC_4_(3) in B-CLL cells after 1- and 3-hour incubation with 80 μM α-BSB was higher than in control (MFI_1h_ = 23 ± 7.8 and MFI_3h_ = 65 ± 13 *vs* MFI_basal_ = 7.5 ± 0.6; *p* < 0.001). This indicated the depolarization of plasma membrane, a further hallmark of the apoptotic process induced by α-BSB (Figure 2B). Finally, loading cells with the Ca^2+^ indicator Fluo-4 AM, an increased Ca^2+^ influx was evident after 20 minutes (Figure 2C). Overall, these results indicated that α-BSB rapidly induced plasma membrane alterations and loss of plasma membrane integrity in B-CLL cells due to apoptosis.

### α-BSB causes loss of mitochondrial transmembrane potential (ΔΨm)

**Figure 3:**
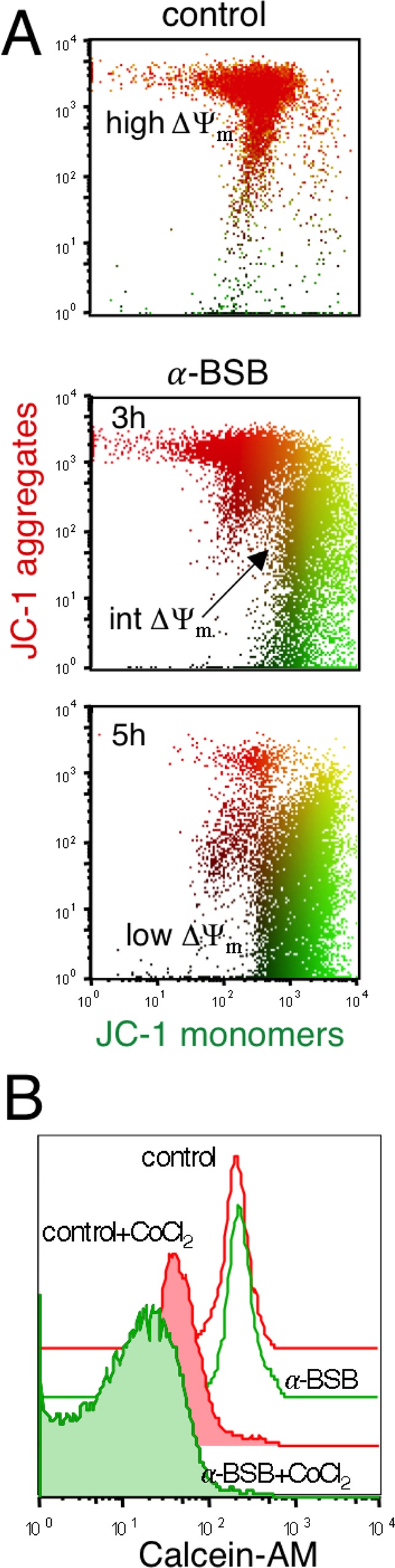
α-BSB caused rapid loss of mitochondria integrity in B-CLL cells Treatment with 40 μM α-BSB for 3 and 5 hours. (**A**) Mitochondrial transmembrane potential (ΔΨm) as evaluated by JC-1 staining. Untreated B-CLL cells presented a typical normal high ΔΨm. Upon treatment, cell fluorescence moved downward (intermediate and low ΔΨm), due to progressive JC-1 dislocation from mitochondria to cytosol. Data are presented by PolyChromatic plots. (**B**) Calcein-AM assay demonstrated the impairment of mPTP function. After 5 hours of treatment, cells were loaded with calcein-AM and MFI was similar to that of untreated cells. After adding CoCl_2_ MFI was lower in treated than in untreated cells (*p* < 0.01), indicating that CoCl_2_ entered damaged mitochondria and quenched calcein inside them. Each shown experiment is representative of 5.

We have previously demonstrated by 5,5′,6,6′-tetra-chloro-1,1′,3,3′-tetra-ethyl-benz-imidazolyl-carbo-cyanine iodide (JC-1) staining that α-BSB dissipates the ΔΨ_m_ in a variety of cell types [31]. Here we investigated the ΔΨ_m_ in α-BSB-treated B-CLL cells. Figure 3A shows that exposure to α-BSB led well-polarized mitochondria to progressively lose their JC-1-dependent red fluorescence, shifting downward, which indicated ΔΨ_m_ dissipation. The calcein-acetoxymethyl ester (calcein-AM) assay explores the mPTP opening, an early event in the damaged cells. Non-fluorescent calcein-AM become fluorescent in cells after cleavage of AM groups *via* non-specific esterase activity in the cytosol and mitochondria. CoCl_2_ cannot enter into healthy mitochondria when the mPTP is closed and it quenches cytoplasmic fluorescence leaving unmodified the mitochondrial one. Figure 3B shows cells that were treated with α-BSB and then loaded with calcein-AM. MFI in treated and untreated cells was 244.5 ± 38 *vs* 253 ± 17, respectively, but after adding CoCl_2_, MFI was 15.8 ± 5 *vs* 38.2 ± 3.8, respectively (*p* < 0.01) due to the quenching of both cytoplasmic and mitochondrial fluorescence in treated cells, as a consequence of the mPTP irreversible opening. Therefore, α-BSB disrupted the membrane permeability of mitochondria, induced ΔΨ_m_ dissipation and triggered the apoptotic death of B-CLL cells.

### α-BSB induces pores in lysosomal membrane

**Figure 4:**
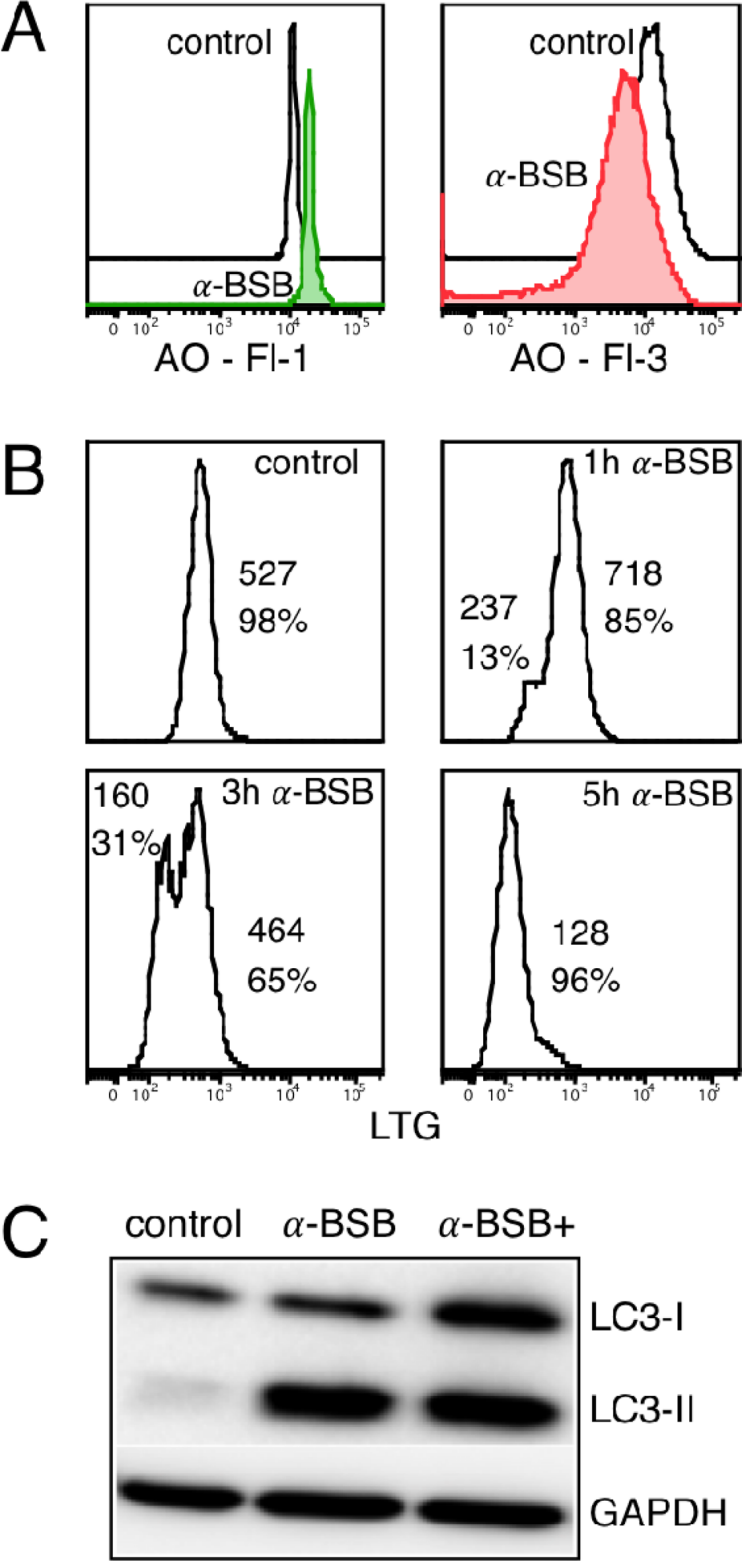
α-BSB induced lysosomal membrane injury and blocked autophagic flux in B-CLL cells Treatment with 80 μM α-BSB for 3 hours. (**A**) Left side: AO-relocation assay. Cells were stained with acridine orange (AO) and treated. Leakage of lysosomal AO content into the cytoplasm was detected by flow cytometry as an increase of green fluorescence (FI-1). Right side: AO-uptake assay. Cells were stained with AO. Impaired lysosomal dye accumulation was measured as a reduction in red fluorescence (Fl-3). (**B**) Kinetics of lysosomal perturbation by LTG-uptake assay. Cells with higher MFI within 1 hour of treatment and time-dependent appearance of an incremental population of dimly fluorescent cells are depicted. These findings are compatible with an enlargement and subsequent collapse of lysosomes. The values of MFI and the corresponding percentage of cells are reported. (**C**) Expression levels of LC3-I, LC3-II and GAPDH in B-CLL cells untreated (control) or treated for 16 hours with α-BSB alone or in association with pepstatin A and E-64D (α-BSB+): no further accumulation of α-BSB+ LC3-II band. Each shown experiment is representative of 5.

The effect of α-BSB on lysosomes was studied using the fluorescent lysosomotropic dyes acridine orange (AO) and LysoTracker Green DND-26 (LTG). AO is a metachromatic fluorophore that becomes charged and retained by proton trapping within the lysosomal compartment. When cells are excited by blue light (488 nm blue laser in flow cytometry), lysosomes emit intense red fluorescence while the cytosol and nuclei show weak diffuse green fluorescence. Cells with a reduced number of intact AO-accumulating lysosomes are pale. When lysosomes are damaged, AO is released to the cytosol in monomeric form and it turns green [43, 44]. The AO-relocation method [44–47] evaluates the degree of lysosomal damage based on the measure of cytosolic green fluorescence. Instead, the AO-uptake method measures directly the lysosomal red fluorescence. In the α-BSB-treated B-CLL cells, the AO-relocation assay demonstrated an increase in green fluorescence detectable up to 3 hours after treatment (Figure 4A, left), followed by a decrease, likely due to the extracellular release of cytoplasmic AO, secondary to plasma membrane permeabilization. The AO-uptake assay demonstrated a decrease in red fluorescence (Figure 4A, right). Notably, this was further confirmed by the LTG-uptake assay aimed to evaluate the lysosomal activity [48, 49]. Under baseline conditions, LTG was located at large vesicles in the cytoplasm. Treatment induced a time-dependent appearance and increment of a population of dimly fluorescent cells (Figure 4B).

### Autophagic flux inhibition

Since lysosomes were damaged by α-BSB treatment, we asked whether α-BSB promoted some impairment of the autophagic process, which plays a role in B-CLL cells survival [50]. Autophagy is a catabolic process during which cytoplasmic organelles and proteins are nonselectively degraded to rescue cells from death. Double-membranous structures emerge in the cytosol forming an autophagosome that sequesters cytoplasmic proteins and organelles. If the rescue fails, apoptosis is triggered. During autophagy, a cytosolic form of the microtubule-associated protein light chain 3 (LC3-I) is conjugated to phosphatidylethanolamine to form LC3-phosphatidylethanolamine (LC3-II). This latter is coupled to autophagosomal membranes. The autophagosome then fuses with lysosomes to form autolysosomes, intra-autophagosomal components are broken down by hydrolases and LC3-II is degraded. Hence, LC3-II is an autophagosomal marker for monitoring autophagic activity by immunoblotting or other methods. In evaluating autophagy by LC3-II immunoblotting technique, we observed that α-BSB induced an increment of LC3-II band in B-CLL cells. The increment may indicate either upregulation of autophagosome formation or blockage of autophagic degradation [51–53]. Figure 4C shows no further accumulation of LC3-II in the presence of pepstatin A and E-64D, inhibitors of the degradation of the autophagic cargo inside the autophagosome. Overall, and similarly to pepstatin or E-64D, α-BSB acts by blocking the autophagic degradation, in keeping with the observed cumulative damage to lysosomal membrane shown in Figure 4A–4B. Therefore, immunoblotting studies of the autophagic process support that α-BSB is an antiautophagic agent that causes the irreversible block of the autophagic flux.

## DISCUSSION

We and others have previously shown that the natural sesquiterpene alcohol α-BSB induces apoptotic death of a variety of neoplastic cells either *in vitro* or in animal models [31–33, 54–58]. Notably, we have demonstrated its cytotoxicity against lymphoblastic, myeloblastic and chronic myeloid leukemia cells in preclinical studies [22, 31, 32]. Our present findings show that α-BSB induces efficient lysis of primary B-CLL. The neoplastic CD5/CD19 B cells resulting sensitive to α-BSB were obtained from 45 untreated patients representative of the typical diagnostic characteristics, natural history and clinical outcome of the B-CLL (Table 1). In parallel and in each patient, we analyzed both leukemic cells and normal residual CD5-negative B cells, monocytes and T cells. Our main results may be summarized as follows. *a)* α-BSB preferentially induced apoptosis of leukemic cells (Figure 1A–1C). *b)* Concurrently, it had a clear-cut antiautophagic potential (Figure 4). As α-BSB could both promote apoptosis and inhibit autophagy, we may also refer to α-BSB as a dual (proapoptotic, antiautophagic) agent. *c)* Though at a lesser degree in comparison to leukemic cells, α-BSB had some cytotoxic effect also against normal residual B cells, monocytes and even T cells, which can also account for a plain anti-inflammatory/anti-microenvironment activity (Figure 1A). The differential amounts of lipid raft could possibly help explain the cell-type selectivity (Figure 1C).

We must place these results in the frame of the natural and therapeutic history of B-CLL. This leukemia has been characterized by a substantial lack of effective treatments until the 1990s. Alkylating agents were the only available therapeutic tools, no more than a symptomatic approach to downsize the neoplastic mass, when needed by symptoms, with no success in inducing disease remission or improving patients' survival [25]. Hence, treatment was preferentially delayed until disease-related symptoms occurred, an approach compatible with the usually indolent clinical course of B-CLL [1, 24, 59]. The introduction of purine analogue-based immuno-chemotherapy incorporating anti-CD20 antibodies was the turning-point that over the last 20 years greatly improved B-CLL treatment in terms of disease remission, failing, however, definitive cure [3, 24–27]. The development of an array of phosphokinase inhibitors was a further, more recent, improvement grounded in a better knowledge of the transduction network downstream BCR, chemokine receptors, receptors for ligands expressed by a variety of microenvironment cells (including monocytes, T lymphocytes, stromal cells etc.), and other receptors on B-CLL cells (including CD19, CD5, CD38 etc.). These agents are administered orally, are often successful even in B-CLL cells bearing unfavorable genetic mutations, and display significant less toxicity than immuno-chemotherapy [23–26].

The natural history and the pattern of response to the treatment of B-CLL reflect perhaps the degree of microenvironmental pleiotropic integration of normal mature B lymphocytes [3, 13, 14, 16–20, 60]. Though we do not completely understand what processes belong to the basic instruction set of B lymphocytes, it is quite clear that their neoplastic counterpart still respond to strong microenvironmental signals while surviving some death checkpoints, which seem to have lost efficacy: *1)* the apoptosis/autophagy balance favors survival, *2)* the mitotic checkpoints restraining cell cycle are not efficient and *3)* membrane receptors regulating responses to microenviroment-related signals seem to be frozen in an activated configuration that confers a continuous streaming of signaling [20, 60].

*1)* In the context of apoptosis/autophagy balance, a plausible explanation for the resistance of B-CLL cells to eradication is supported by their autophagic potential, which probably upon treatment allows for the persistency of reservoirs of neoplastic stem cells, in a way somehow similar to that observed for chronic myeloid leukemia CD34 cells [32, 61]. Therefore, the dual proapoptotic, antiautophagic activity of α-BSB [22] is a point whorthy of interest in medical as well as biological terms, based on the fact that autophagy can rescue from drug-induced death at least some specific type of cancer cells. In particular, a great deal of attention has been paid to the role of autophagy in rescuing cancer stem cells upon treatment, resulting in the therapy-resistance of this neoplastic compartment, which in turn results in the failure of cancer eradication and subsequent possible clinical relapse. It has been shown that the blocking of autophagy allows for the eradication of the residual neoplastic disease [61]. Moreover, since α-BSB participates to both the induction and inhibition of autophagy while inducing apoptosis [22, 31, 32], it seems reasonable to situate α-BSB action at the core molecular crossroads regulating both apoptotic and autophagic pathways. The dual action might involve two different layers of control: (a) the α-BSB interaction with the BH3-only protein BID [33], which may deliver signals through modulation of BCL2-Beclin1 interactions at the endoplasmic reticulum leading to Beclin1-dependent autophagy [62] and BID-dependent apoptosis [22, 33]; (b) the α-BSB-dependent induction of pores in lysosomal membranes and the collapse of the lysososmal compartment (Figure 4A–4B), which neutralizes the autophagosome function and inhibits the autophagic flux (Figure 4C). Therefore, the core level machinery regulating the crosstalk between autophagy and apoptosis that is targeted and thus highlighted by α-BSB-induced phenomena may lead on the one hand to a better understanding of the role of BCL2-family molecules in oncogenesis and tumor progression, while on the other hand it may represent a target for refined therapeutic tools, such as α-BSB itself and other agents that can affect the apoptosis/autophagy balance [22–24]. Besides, the possible selective degradation of mitochondria through autophagy, which also occurs, adds further complexity [63]. *2)* If we focus on the cell cycle disregulation, it follows from the above considerations that α-BSB through its cytostatic activity as well as its proapoptotic potential may be expected to overcome the unrestrained cycling of tumor cells in typical indolent forms of B-CLL as well as in more aggressive forms of B-CLL or in more aggressive subclones arising inside the mainly indolent B-CLL cell population. *3)* If we take the leukemia/microenvironment crosstalk into consideration, our findings suggest that α-BSB may exert pleiotropic activities, inducing both efficient direct B-CLL death and microenvironmental modulation. This latter has at least two facets. First, α-BSB may account for an effect able to hamper the microenvironment-related cell-to-cell signals, especially from T4 cells, supporting B-CLL cell survival [64]. Second, the pathogenetic role of the peritumoral inflammatory responses and, therefore, the potential therapeutic significance of targeting them has been variously highlighted over the recent years [11, 15, 19, 20, 65, 66]; in this context, α-BSB may counteract in an autacoid-like manner the B-, T- and monocyte-dependent pro-inflammatory responses, known to enhance leukemic growth [67]. Consequently, the effect of α-BSB on B-CLL is expected to increase over time, ranging from direct cytotoxicity to the maintenance of a microenvironment less favorable to B-CLL growth on the long run, a relevant impact for an agent eligible to be chronically administered. In that respect, α-BSB merits some additional interest on basic clinical grounds such as no or low general toxicity, easy oral delivering and affordable cost. [31, 32, 68, 69].

But at the very heart of our mechanistic views on α-BSB lies a broad biologic issue worthy of consideration. Previous studies have shown that the cytotoxic activity of α-BSB interests a variety of neoplastic cell types [31–33, 54–56, 58], which could indicate an agent targeting basic and quite ancient cellular biochemical pathways, and, according to our findings, the evidence corroborates that α-BSB is an example of xenohormesis involving the function of BH3-only domain BCL2-family proteins at the crossroad of apoptosis and autophagy pathways [22, 62, 70], though the fine biochemical details of the apoptosis/autophagy crossmachinery targeted by α-BSB remain elusive. Indeed, there is no surprise that these pathways are shared between different types of cancers including leukemias. However, since leukemias, namely acute leukemias, are the most sensitive targets for α-BSB [22, 31, 32], this likely points to a specific relevance for the evolutionarily conserved apoptosis/autophagy crosstalk machinery in hematopoietic mesodermal-derived lineages, and, if we can learn the lesson from the neoplastic counterpart, precisely where leukemia cells arise. We argument that in these cells exclusive unilineage adoption is barred by fusion transcription factors that maintain cells perpetually looping at a low pace without differentiation [64, 71–73]. As a result their apoptosis/autophagy machinery is strongly unbalanced towards the autophagic rescue. This allow for the maintenance of an early leukemic compartment highly resistant to eradication, except perhaps for agents targeting autophagy.

In conclusion, we provide preclinical evidence that the sesquiterpene α-BSB can promote proapoptotic and antiautophagic activity in B-CLL cells, leading to efficient lysis. Simultaneously, α-BSB has some lytic activity on normal B-lymphocytes, monocytes and T cells, which may entail an overall damping effect against microenvironment components that sustain leukemic cell growth.

## MATERIALS AND METHODS

### Patents' cells and ethical requirements

Viable peripheral blood mononuclear cells (PBMC) of 45 patients with untreated B-CLL were obtained as previously described on a Ficoll-Hypaque gradient from peripheral blood [74, 75]. The diagnosis of B-CLL was made according to the current guidelines [24] as previously described [10] and fulfilled diagnostic criteria for common B-CLL at the Hematology Section of the Department of Medicine, University of Verona (Verona, Italy), starting from 2002. The study was performed in the context of the project 1828/2010 approved by the ethics committee of the Verona University Hospital and a written informed consent was obtained according to law.

### Cytotoxicity assay

Cells resuspended in RPMI-1640 (Invitrogen, Carlsbad, CA), supplemented with 10% heat-inactivated fetal bovine serum (Invitrogen), 50 U/mL penicillin and 50 μg/mL streptomycin (complete medium, CM), seeded at a density of 1 × 10^5^ cells/mL in 24-well plates and incubated at 37°C in 5% CO_2_ were exposed for 24 hours to 20, 40, 80 μM of α-BSB (dissolved in DMSO 1:40; derived from natural organic compounds, purity ≥93%; Sigma-Aldrich, St. Louis, MO), representing the calculated soluble fraction in the assay as reported elsewhere [31]. At the end of the culture, the absolute counts of normal and leukemic leukocytes sub-populations were measured with TruCOUNT tubes (Becton Dickinson, San Jose, CA) by flow cytometry according to the manufacturer's instructions with minor modifications. Briefly, 200 μL samples, a mixture of moAbs (CD45-APC-H7, CD5-APC, CD19-PE, CD3-FITC) and 7-amino-actinomycin D for dead cells exclusion (all reagents from Becton Dickinson) were added to the TruCOUNT tubes. After a 15-minute incubation at room temperature, 1 mL lysing reagent (Biosource, Nivelles, BE) was added for 10 minutes. A total of 40,000 beads were acquired on a FACSCanto cytometer (Becton Dickinson) and analyzed by FlowJo 9.3.3 software (Tree Star, Ashland, OR) as Dot Plot or PolyChromatic Plot. Hierarchical gating was used to accurately enumerate different populations. Data were expressed as ratio of number of cells treated with α-BSB to number of cells treated with vehicle alone.

### Lipid raft component quantification

Cells were prestained for 10 minutes at room temperature with a mix of moAbs specific for different leukocyte populations (CD3-PerCP, CD5-APC, CD19-APC-H7) and, after washing, the main components of LRs (ganglioside GM1, Flotillin-1 and cholesterol) were quantified. 1. GM1. Cells were incubated at 4°C for 10 minutes with Alexa Fluor 488 cholera toxin subunit B (CT-B) (Invitrogen) which specifically binds to the pentasaccharide chain of GM1. Cells were then washed 3 times with PBS and analyzed in flow cytometry. 2. Flotillin-1. Cells fixed and permeabilized by a commercial kit (eBiosciences, San Diego, CA) according to the manufacturer's instructions were incubated with an anti-Flotillin-1-Cy3 antibody (Sigma) for 1 hour at 4°C. After washing samples were analyzed in flow cytometry. 3. Cholesterol. Cells fixed with component A of Fix&Perm kit (AnDerGrub Bio Research, Kaumberg, AT) were incubated with 100 μg/mL Filipin III (Sigma) for 1 hour at room temperature. After washing samples were analyzed in flow cytometry [37]. GM1, Flotillin-1 and cholesterol data were expressed in arbitrary units as the ratio between median fluorescense intensity of stained cells (subtracted of that of unstained ones) and median forward scatter (as a surrogate of cell dimension) multiplied by 10^4^.

### Evaluation of plasma membrane damage

1. Apoptosis and membrane permeability. Cells were treated for 1 to 3 hours with 80 μM α-BSB, washed with PBS and stained with Annexin-V-FITC (Miltenyi Biotec, Bergisch Gladbach, DE) for 15 minutes and TO-PRO-3 (Invitrogen) immediately before acquisition. 2. Membrane potential. Cells were treated for 1 to 3 hours with 80 μM α-BSB, incubated with 100 nM DiBAC_4_(3) (Invitrogen) for 20 minutes and analyzed by flow cytometry as MFI. 3. Ca^2+^ influx. Cells were resuspended in HBSS/Ca^2+^ and loaded with 2 μM Fluo-4 acetoxymethyl ester (Fluo-4 AM, Invitrogen) for 45 minutes at 37°C. After washing cells were incubated for 10, 20, 30 and 40 minutes with 80 μM α-BSB. Time-dependent cells fluorescence was recorded by flow cytometry.

### Mitochondrial membrane damage assays

Cells resuspended in CM at 1 × 10^6^/mL were treated with 40 μM α-BSB for 3 and 5 hours at 37°C.

1. Mitochondrial transmembrane potential (ΔΨm). As previously described [31] cells were washed with pre-warmed CM, loaded with 4 μM JC-1 (Molecular Probes, Eugene, OR) for 30 minutes then washed twice with PBS. Aliquots of each sample were resuspended in PBS and analyzed by flow cytometry. Visualization of JC-1 monomers and JC-1 aggregates was done using filter sets for fluorescein and rhodamine dyes. Image analysis was done by using Axiovision 3 software. The other aliquot of each sample was resuspended in PBS and analyzed by flow cytometry. 2. Mitochondrial permeability transition pore (mPTP). Cells were washed, resuspended in HBSS/Ca^2+^, loaded with 10 nM calcein-AM with or without 400 μM CoCl_2_ for 15 minutes at 37°C (MitoProbe Transition Pore assay kit, Invitrogen) and analyzed by flow cytometry.

### Lysosome injury assays

1. Acridine orange-relocation assay. Cells stained with 0,5 μg/mL AO (Molecular Probes) at 37°C for 15 minutes, washed twice, treated with 80 μM α-BSB for 3 hours, and analized by flow cytometry (FL-1 channel). 2. AO-uptake assay. Cells incubated with 80 μM α-BSB for 3 hours were stained with 0,5 μg/mL AO for 15 minutes at 37°C, washed twice and analized by flow cytometry (FL-3 channel). 3. LysoTracker green-uptake assay. Cells were incubated with 80 μM α-BSB for 1, 3 and 5 hours. Then they were washed and stained at 37°C for 1 hour with 75 nM of the acidotropic dye LTG (Molecular Probes) and analized by flow cytometry (FL-1 channel).

### Detection of autophagic flux

5 × 10^6^ cells were treated with either DMSO (vehicle) or 80 μM α-BSB for 16 hours. In order to inhibit the degradation of autophagic cargo, Pepstatin A (Sigma, 10 μg/mL) and E-64D (Sigma, 10 μg/mL) were added to the media. Then they were harvested, washed in cold PBS, resuspended in Lysis Buffer (Thermo Scientific, Rockford, IL) plus protease inhibitor cocktail (Sigma). Whole cell lisates were separated on a 14% SDS-PAGE and analyzed by immunoblotting with anti-LC3 antibody and with anti-GAPDH antibody (Cell Signaling Technology, Beverly, MA) and VeriBlot-HRP secondary antibody (Abcam, Cambridge UK). Development was done by enhanced chemiluminescent plus detection reagents (Thermo Scientific) [51].

### Statistics

Continuous variables were summarized as the mean ± SD. Data were summarized by percentages when appropriate. Student's *t*-test for means, chi-squared tests, the Mann–Whitney *U* test, and Kruskall–Wallis analysis of variance (ANOVA) by ranks were used, as appropriate and were considered significant for *p* values *<* 0.05. Data were analyzed using the statistical software Stata 12.1 (www.stata.com).

## Abbreviations

B-CLL: B chronic lymphocytic leukemia
α-BSB: α-bisabolol
CM: complete medium
moAbs: monoclonal antibodies
LRs: lipid rafts
GM1: monosialotetrahexosylganglioside
CT-B: cholera toxin B
DiBAC_4_: Bis-(1,3-Dibutylbarbituric Acid) Trimethin Oxonol
MFI: mean fluorescence intensity
JC-1: 5,5′,6,6′-tetra-chloro-1,1′,3,3′--tetra-ethyl-benz-imidazolylcarbo-cyanine iodide
ΔΨm: mitochondrial transmembrane potential
mPTP: mitochondrial permeability transition pore
AO: acridine orange
LTG: LysoTracker Green DND-26
LC3-I: microtubule-associated protein light chain 3
LC3-II: LC3-phosphatidylethanolamine
TO-PRO-3: Quinolinium, 4-[3-(3-methyl-2(3H)-benzothiazolylidene)-1-propenyl]-1-[3-(trimethylammonio)propyl]-, diiodide

## References

[1] O'Brien S, del Giglio A, Keating M (1995). Advances in the biology and treatment of B-cell chronic lymphocytic leukemia. Blood.

[2] Schroeder HW, Dighiero G (1994). The pathogenesis of chronic lymphocytic leukemia: analysis of the antibody repertoire. Immunol Today.

[3] Grever MR, Lucas DM, Dewald GW, Neuberg DS, Reed JC, Kitada S, Flinn IW, Tallman MS, Appelbaum FR, Larson RA, Paietta E, Jelinek DF, Gribben JG (2007). Comprehensive assessment of genetic and molecular features predicting outcome in patients with chronic lymphocytic leukemia: results from the US Intergroup Phase III Trial E2997. J Clin Oncol.

[4] Fais F, Ghiotto F, Hashimoto S, Sellars B, Valetto A, Allen SL, Schulman P, Vinciguerra VP, Rai K, Rassenti LZ, Kipps TJ, Dighiero G, Schroeder HW (1998). Chronic lymphocytic leukaemia B cells express restricted sets of mutated and unmutated antigen receptors. J Clin Invest.

[5] Damle RN, Wasil T, Fais F, Ghiotto F, Valetto A, Allen SL, Buchbinder A, Budman D, Dittmar K, Kolitz J, Lichtman SM, Schulman P, Vinciguerra VP (1999). Ig V gene mutation status and CD38 expression as novel prognostic indicators in chronic lymphocytic leukemia. Blood.

[6] Hamblin TJ, Davis Z, Gardiner A, Oscier DG, Stevenson FK (1999). Unmutated Ig V(H) genes are associated with a more aggressive form of chronic lymphocytic leukaemia. Blood.

[7] Lanham S, Hamblin T, Oscier D, Ibbotson R, Stevenson F, Packham G (2003). Differential signaling via surface IgM is associated with VH gene mutational status and CD38 expression in chronic lymphocytic leukemia. Blood.

[8] Palamarchuk A, Efanov A, Nazaryan N, Santanam U, Alder H, Rassenti L, Kipps T, Croce CM, Pekarsky Y (2010). 13q14 deletions in CLL involve cooperating tumor suppressors. Blood.

[9] Chen L, Widhopf G, Huynh L, Rassenti L, Rai KR, Weiss A, Kipps TJ (2002). Expression of ZAP-70 is associated with increased B-cell receptor signaling in chronic lymphocytic leukemia. Blood.

[10] Montresor A, Bolomini-Vittori M, Simon SI, Rigo A, Vinante F, Laudanna C (2009). Comparative analysis of normal versus CLL B-lymphocytes reveals patient-specific variability in signaling mechanisms controlling LFA-1 activation by chemokines. Cancer Res.

[11] Montresor A, Toffali L, Mirenda M, Rigo A, Vinante F, Laudanna C (2015). JAK2 tyrosine kinase mediates integrin activation induced by CXCL12 in B-cell chronic lymphocytic leukemia. Oncotarget.

[12] Trentin L, Agostini C, Facco M, Piazza F, Perin A, Siviero M, Guerrieri C, Galvan S, Adami F, Zambello R, Semenzato G (1999). The chemokine receptor CXCR3 is expressed on malignant B cells and mediates chemotaxis. J Clin Invest.

[13] Panayiotidis P, Jones D, Ganeshaguru K, Foroni L, Hoffbrand AV (1996). Human bone marrow stromal cells prevent apoptosis and support the survival of chronic lymphocytic leukaemia cells in vitro. Br J Haematol.

[14] Lagneaux L, Delforge A, Bron D, De Bruyn C, Stryckmans P (1998). Chronic lymphocytic leukemic B cells but not normal B cells are rescued from apoptosis by contact with normal bone marrow stromal cells. Blood.

[15] Rigo A, Gottardi M, Zamò A, Mauri P, Bonifacio M, Krampera M, Damiani E, Pizzolo G, Vinante F (2010). Macrophages may promote cancer growth via a GM-CSF/HB-EGF paracrine loop that is enhanced by CXCL12. Mol Cancer.

[16] Burger JA, Burger M, Kipps TJ (1999). Chronic lymphocytic leukemia B cells express functional CXCR4 chemokine receptors that mediate spontaneous migration beneath bone marrow stromal cells. Blood.

[17] Mohle R, Failenschmid C, Bautz F, Kanz L (1999). Overexpression of the chemokine receptor CXCR4 in B cell chronic lymphocytic leukemia is associated with increased functional response to stromal cell-derived factor-1 (SDF-1). Leukemia.

[18] Gehrke I, Gandhirajan RK, Poll-Wolbeck SJ, Hallek M, Kreuzer KA (2011). Bone marrow stromal cell-derived vascular endothelial growth factor (VEGF) rather than chronic lymphocytic leukemia (CLL) cell-derived VEGF is essential for the apoptotic resistance of cultured CLL cells. Mol Med.

[19] Vinante F, Rigo A (2013). Heparin-binding epidermal growth factor-like growth factor/diphtheria toxin receptor in normal and neoplastic hematopoiesis. Toxins (Basel).

[20] van Attekum MH, Eldering E, Kater AP (2017). Chronic lymphocytic leukemia cells are active participants in microenvironmental cross-talk. Haematologica.

[21] Li P, Harris D, Liu Z, Liu J, Keating M, Estrov Z (2010). Stat3 activates the receptor tyrosine kinase like orphan receptor-1 gene in chronic lymphocytic leukaemia cells. PLoS One.

[22] Rigo A, Vinante F (2016). The antineoplastic agent α-bisabolol promotes cell death by inducing pores in mitochondria and lysosomes. Apoptosis.

[23] Vogler M, Walter HS, Dyer MJS (2017). Targeting anti-apoptotic BCL2 family proteins in haematological malignancies - from pathogenesis to treatment. Br J Haematol.

[24] Hallek M, Pflug N (2011). State of the art treatment of chronic lymphocytic leukaemia. Blood Rev.

[25] Routledge DJ, Bloor AJ (2016). Recent advances in therapy of chronic lymphocytic leukaemia. Br J Haematol.

[26] Butler LA, Tam CS, Seymour JF (2017). Dancing partners at the ball: Rational selection of next generation anti-CD20 antibodies for combination therapy of chronic lymphocytic leukemia in the novel agents era. Blood Rev.

[27] Cartron G, Watier H (2017). Obinutuzumab: what is there to learn from clinical trials?. Blood.

[28] Ren Y, Yu J, Kinghorn AD (2016). Development of Anticancer Agents from Plant-Derived Sesquiterpene Lactones. Curr Med Chem.

[29] Klayman DL (1985). Qinghaosu (artemisinin): An antimalarial drug from China. Science.

[30] Wang X, Zhang C, Yan X, Lan B, Wang J, Wei C, Cao X, Wang R, Yao J, Zhou T, Zhou M, Liu Q, Jiang B (2016). A novel bioavailable BH3 mimetic efficiently inhibits colon cancer via cascade effects of mitochondria. Clin Cancer Res.

[31] Cavalieri E, Rigo A, Bonifacio M, Carcereri de Prati A, Guardalben E, Bergamini C, Fato R, Pizzolo G, Suzuki H, Vinante F (2011). Pro-apoptotic activity of α-bisabolol in preclinical models of primary human acute leukemia cells. J Transl Med.

[32] Bonifacio M, Rigo A, Guardalben E, Bergamini C, Cavalieri E, Fato R, Pizzolo G, Suzuki H, Vinante F (2012). α-bisabolol is an effective proapoptotic agent against BCR-ABL(+) cells in synergism with imatinib and nilotinib. PLoS One.

[33] Darra E, Abdel-Azeim S, Manara A, Shoji K, Marechal JD, Mariotto S, Cavalieri E, Perbellini L, Pizza C, Perahia D, Crimi M, Suzuki H (2008). Insight into the apoptosis-inducing action of alpha-BSB towards malignant tumor cells: involvement of lipid rafts and Bid. Arch Biochem Biophys.

[34] Patra SK, Bettuzzi S (2007). Epigenetic DNA-methylation regulation of genes coding for lipid raft-associated components: a role for raft proteins in cell transformation and cancer progression. Oncol Rep.

[35] Spiegel S (1988). Insertion of ganglioside GM1 into rat glioma C6 cells renders them susceptible to growth inhibition by the B subunit of cholera toxin. Biochim Biophys Acta.

[36] Fantini J, Maresca M, Hammache D, Yahi N, Delézay O (2000). Glycosphingolipid (GSL) microdomains as attachment platforms for host pathogens and their toxins on intestinal epithelial cells: activation of signal transduction pathways and perturbations of intestinal absorption and secretion. Glycoconj J.

[37] Meyer zum Büschenfelde C, Feuerstacke Y, Götze KS, Scholze K, Peschel C (2008). GM1 expression of non-Hodgkin's lymphoma determines susceptibility to rituximab treatment. Cancer Res.

[38] Langhorst MF, Reuter A, Stuermer CA (2005). Scaffolding microdomains and beyond: the function of reggie/flotillin proteins. Cell Mol Life Sci.

[39] Stuermer CA, Plattner H (2005). The ‘lipid raft’ microdomain proteins reggie-1 and reggie-2 (flotillins) are scaffolds for protein interaction and signalling. Biochem Soc Symp.

[40] Fernow I, Icking A, Tikkanen R (2007). Reggie-1 and reggie-2 localize in non-caveolar rafts in epithelial cells: cellular localization is not dependent on the expression of caveolin proteins. Eur J Cell Biol.

[41] Neumann-Giesen C, Fernow I, Amaddii M, Tikkanen R (2007). Role of EGF-induced tyrosine phosphorylation of reggie-1/flotillin-2 in cell spreading and signaling to the actin cytoskeleton. J Cell Sci.

[42] Muller CP, Stephany DA, Winkler DF, Hoeg JM, Demosky SJ, Wunderlich JR (1984). Filipin as a flow microfluorometry probe for cellular cholesterol. Cytometry.

[43] Bradley DF, Wolf MK (1959). Aggregation of dyes bound to polyanions. Proc Natl Acad Sci U S A.

[44] Zdolsek JM, Olsson GM, Brunk UT (1990). Photooxidative damage to lysosomes of cultured macrophages by acridine orange. Photochem Photobiol.

[45] Servais H, Van Der Smissen P, Thirion G, Van Der Essen G, Van Bambeke F, Tulkens PM, Mingeot-Leclercq MP (2005). Gentamicin-induced apoptosis in LLC-PK1 cells: Involvement of lysosomes and mitochondria. Toxicol Appl Pharmacol.

[46] Zareba M, Raciti MW, Henry MM, Sarna T, Burke JM (2006). Oxidative stress in ARPE-19 cultures: Do melanosomes confer cytoprotection?. Free Radic Biol Med.

[47] Olsson GM, Rungby J, Rundquist I, Brunk UT (1989). Evaluation of lysosomal stability in living cultured macrophages by cytofuorometry. Effect of silver lactate and hypotonic conditions. Virchows Arch B Cell Pathol Incl Mol Pathol.

[48] Arsham AM, Neufeld TP (2009). A genetic screen in Drosophila reveals novel cytoprotective functions of the autophagy-lysosome pathway. PLoS One.

[49] Yoon J, Kim KJ, Choi YW, Shin HS, Kim YH, Min J (2010). The dependence of enhanced lysosomal activity on the cellular aging of bovine aortic endothelial cells. Mol Cell Biochem.

[50] Rosati A, Basile A, Falco A, d'Avenia M, Festa M, Graziano V, De Laurenzi V, Arra C, Pascale M, Turco MC (2012). Role of BAG3 protein in leukemia cell survival and response to therapy. Biochim Biophys Acta.

[51] Mizushima N (2004). Methods for monitoring autophagy. Int J Biochem Cell Biol.

[52] Mizushima N, Yoshimori T (2007). How to interpret LC3 immunoblotting. Autophagy.

[53] Klionsky DJ, Abdelmohsen K, Abe A, Abedin MJ, Abeliovich H, Acevedo Arozena A, Adachi H, Adams CM, Adams PD, Adeli K, Adhihetty PJ, Adler SG, Agam G (2016). Guidelines for the use and interpretation of assays for monitoring autophagy (3rd edition). Autophagy.

[54] Seki T, Kokuryo T, Yokoyama Y, Suzuki H, Itatsu K, Nakagawa A, Mizutani T, Miyake T, Uno M, Yamauchi K, Nagino M (2011). Antitumor effect of α-bisabolol against pancreatic cancer. Cancer Sci.

[55] Costarelli L, Malavolta M, Giacconi R, Cipriano C, Gasparini N, Tesei S, Pierpaoli S, Orlando S, Suzuki H, Perbellini L, Piacenza F, Emanuelli M, Mocchegiani E (2010). In vivo effect of α-bisabolol, a non toxic sesquiterpene alcohol, on the induction of spontaneous mammary tumors in HER-2/neu transgenic mice. Oncol Res.

[56] Cavalieri E, Mariotto S, Fabrizi C, de Prati AC, Gottardo R, Leone S, Berra LV, Lauro GM, Ciampa AR, Suzuki H (2004). α-Bisabolol, a nontoxic natural compound, strongly induces apoptosis in glioma cells. Biochem Biophys Res Commun.

[57] Cavalieri E, Bergamini C, Mariotto S, Leoni S, Perbellini L, Darra E, Suzuki H, Fato R, Lenaz G (2009). Involvement of mitochondrial permeability transition pore opening in α-bisabolol induced apoptosis. FEBS J.

[58] Chen W, Hou J, Yin Y, Jang J, Zheng Z, Fan H, Zou G (2010). α-Bisabolol induces dose- and time-dependent apoptosis in HepG2 cells via a Fas- and mitochondrial-related pathway, involves p53 and NFkappaB. Biochem Pharmacol.

[59] Wendtner CM, Gregor M (2018). Current perspectives on the role of chemotherapy in chronic lymphocytic leukemia. Leuk Lymphoma.

[60] Herman SE, Wiestner A (2016). Preclinical modeling of novel therapeutics in chronic lymphocytic leukemia: the tools of the trade. Semin Oncol.

[61] Mitchell R, Hopcroft LEM, Baquero P, Allan EK, Hewit K, James D, Hamilton G, Mukhopadhyay A, O'Prey J, Hair A, Melo JV, Chan E, Ryan KM (2017). Targeting BCR-ABL-Independent TKI Resistance in Chronic Myeloid Leukemia by mTOR and Autophagy Inhibition. J Natl Cancer Inst.

[62] Kroemer G, Levine B (2008). Autophagic cell death: the story of a misnomer. Nat Rev Mol Cell Biol.

[63] Yamashita SI, Jin X, Furukawa K, Hamasaki M, Nezu A, Otera H, Saigusa T, Yoshimori T, Sakai Y, Mihara K, Kanki T (2016). Mitochondrial division occurs concurrently with autophagosome formation but independently of Drp1 during mitophagy. J Cell Biol.

[64] Sun C, Wiestner A (2017). CLL kinetics in the tumor microenvironment. Oncotarget.

[65] Mantovani A, Marchesi F, Malesci A, Laghi L, Allavena P (2017). Tumour-associated macrophages as treatment targets in oncology. Nat Rev Clin Oncol.

[66] Rigo A, Gottardi M, Damiani E, Bonifacio M, Ferrarini I, Mauri P, Vinante F (2012). CXCL12 and [N33A]CXCL12 in 5637 and HeLa cells: regulating HER1 phosphorylation via calmodulin/ calcineurin. PLoS One.

[67] Sulciner ML, Serhan CN, Gilligan MM, Mudge DK, Chang J, Gartung A, Lehner KA, Bielenberg DR, Schmidt B, Dalli J, Greene ER, Gus-Brautbar Y, Piwowarski J (2017). Resolvins suppress tumor growth and enhance cancer therapy. J Exp Med.

[68] Marjanovic G (2017). The use of inexpensive broad spectrum lower toxicity therapeutics in chronic lymphocytic leukemia. J BUON.

[69] Rowswell-Turner RB, Barr PM (2017). Treatment of chronic lymphocytic leukemia in older adults. J Geriatr Oncol.

[70] Howitz KT, Sinclair DA (2008). Xenohormesis: sensing the chemical cues of other species. Cell.

[71] Enver T, Greaves M (1998). Loops, Lineage, and Leukemia. Cell.

[72] Murry CE, Keller G (2008). Differentiation of Embryonic Stem Cells to Clinically Relevant Populations: Lessons from Embryonic Development. Cell.

[73] Orkin SH, Zon LI (2008). Hematopoiesis: An Evolving Paradigm for Stem Cell Biology. Cell.

[74] Vinante F, Rigo A, Papini E, Cassatella MA, Pizzolo G (1999). Heparin-binding epidermal growth factor-like growth factor/diphtheria toxin receptor expression by acute myeloid leukemia cells. Blood.

[75] Vinante F, Rigo A, Tecchio C, Morosato L, Nadali G, Ricetti MM, Krampera M, Zanolin E, Locatelli F, Gallati H, Chilosi M, Pizzolo G (1998). Serum levels of p55 and p75 soluble TNF receptors in adult acute leukaemia at diagnosis. Correlation with clinical and biological features and outcome. Br J Haematol.

